# Sp6 and Sp8 Transcription Factors Control AER Formation and Dorsal-Ventral Patterning in Limb Development

**DOI:** 10.1371/journal.pgen.1004468

**Published:** 2014-08-28

**Authors:** Endika Haro, Irene Delgado, Marisa Junco, Yoshihiko Yamada, Ahmed Mansouri, Kerby C. Oberg, Marian A. Ros

**Affiliations:** 1 Instituto de Biomedicina y Biotecnología de Cantabria (CSIC-UC-SODERCAN), Santander, Spain; 2 Laboratory of Cell and Developmental Biology, NIDCR, National Institutes of Health, Bethesda, Maryland, United States of America; 3 Max Planck Institute for Biophysical Chemistry, Department of Molecular Cell Biology, Am Fassberg, Göttingen, Germany; 4 Department of Clinical Neurophysiology, University of Göttingen, Göttingen, Germany; 5 Genome and Stem Cell Center, GENKOK, Erciyes University, Kayseri, Turkey; 6 Department of Pathology and Human Anatomy, Loma Linda University, Loma Linda, California, United States of America; 7 Departamento de Anatomía y Biología Celular, Facultad de Medicina, Universidad de Cantabria, Santander, Spain; National Cancer Institute, United States of America

## Abstract

The formation and maintenance of the apical ectodermal ridge (AER) is critical for the outgrowth and patterning of the vertebrate limb. The induction of the AER is a complex process that relies on integrated interactions among the Fgf, Wnt, and Bmp signaling pathways that operate within the ectoderm and between the ectoderm and the mesoderm of the early limb bud. The transcription factors Sp6 and Sp8 are expressed in the limb ectoderm and AER during limb development. *Sp6* mutant mice display a mild syndactyly phenotype while *Sp8* mutants exhibit severe limb truncations. Both mutants show defects in AER maturation and in dorsal-ventral patterning. To gain further insights into the role Sp6 and Sp8 play in limb development, we have produced mice lacking both Sp6 and Sp8 activity in the limb ectoderm. Remarkably, the elimination or significant reduction in *Sp6;Sp8* gene dosage leads to tetra-amelia; initial budding occurs, but neither *Fgf8* nor *En1* are activated. Mutants bearing a single functional allele of *Sp8* (*Sp6^−/−^;Sp8^+/−^*) exhibit a split-hand/foot malformation phenotype with double dorsal digit tips probably due to an irregular and immature AER that is not maintained in the center of the bud and on the abnormal expansion of *Wnt7a* expression to the ventral ectoderm. Our data are compatible with Sp6 and Sp8 working together and in a dose-dependent manner as indispensable mediators of Wnt/βcatenin and Bmp signaling in the limb ectoderm. We suggest that the function of these factors links proximal-distal and dorsal-ventral patterning.

## Author Summary

In this report we examined the functional roles of *Sp6* and *Sp8* during limb development using compound loss-of-function mutants. *Sp6* and *Sp8*, two members of the *Sp* gene family, are expressed in the limb bud ectoderm and function downstream of WNT/βcatenin signaling for *Fgf8* induction. The analysis of the allelic series shows that the progressive reduction in the dose of Sp6 and Sp8 gene products leads to predictable morphology, from syndactyly, to split hand/foot malformation, oligodactyly, truncation and finally amelia, indicating that these two factors act in a complementary manner. The molecular characterization of the mutant limbs reveal that Sp6/Sp8 are required in a dose-dependent manner for *Fgf8* and *En1* induction, thereby placing them as an important link between the induction of the AER and the establishment of dorsal-ventral patterning during limb development.

## Introduction

The apical ectodermal ridge (AER), a specialized thickened epithelium at the distal edge of the developing limb bud, is a major signaling center for limb development (for a review, see [1]). The AER, through the production of several members of the Fibroblast growth factor (Fgf) family, controls survival, proliferation and appropriate gene expression in the subjacent mesoderm [2]–[5].

The AER is formed through a complex and not completely understood process that starts with the induction of the AER precursor cells that are marked by their expression of *Fgf8*. In the mouse, these precursors are specified in the ventral ectoderm of the early limb bud to progressively compact at the tip of the bud to form the mature AER [6], [7]. The mature AER is a linear and regular band of polystratified (in mouse) and pseudostratified (in chick) epithelium rimming the distal dorsal-ventral boundary of the limb bud. Once the digit primordia have formed, the AER flattens and expression of *Fgfs* ceases, first over the interdigital spaces and later over the digit tips [8]. Cell lineage analysis has demonstrated that the AER is a transitory structure formed by a self-sustaining cell population that is exhausted before birth [9].

Initially, the expression of *Fgf10* in the presumptive limb mesoderm activates the expression of *Fgf8* in the overlying ectoderm through the induction of *Wnt3a* [10]–[13]. An ectodermally active Wnt/βcatenin pathway is required throughout limb development, first for AER induction and then for AER maintenance [14], [15]. The genetic removal of *βcatenin* from the limb ectoderm, before the initiation of *Fgf8* expression, completely prevents limb development while its removal after *Fgf8* expression leads to variable truncations [14], [15]. Another essential pathway involved in AER induction and maintenance is the Bone morphogenetic protein (Bmp) signaling pathway. Similar to Wnt/βcatenin signaling, Bmp signaling is required for AER induction, but paradoxically and in stark contrast to Wnt/βcatenin signaling, it exerts a negative influence on AER maintenance. Thus, when Bmp signaling is abolished from the limb ectoderm prior to AER induction, *Fgf8* is never activated and the AER does not form resulting in amelic phenotypes [16], [17]. However, when Bmp signaling is removed from the limb ectoderm after *Fgf8* and AER induction, the expression of *Fgf8* is prolonged in the AER leading to syndactyly [16]. Bmp signaling has been proposed to act both upstream and downstream of Wnt/βcatenin signaling and, despite intensive study, the interactions between these pathways in the induction and maintenance of the AER remains only partially understood [14], [15].

Very interesting is the connection between the AER and the establishment of dorsal-ventral (DV) patterning [18]. During normal development the position of the mature AER always coincides with the DV boundary. However, it is well known that a normal functional AER can form in the absence of normal DV patterning. For instance, in the *eudiplopodia* chick mutant an extra AER appears within the dorsal ectoderm leading to extra double dorsal limb outgrowth [19]. Also, in the double *Wnt7a;En1* (*Engrailed1*) mutant a virtually normal AER forms despite disturbed DV patterning [20], [21]. It has been suggested that the coordination between the position of the AER and the DV boundary depends on BMP signaling because, besides its above mentioned role on AER induction and maintenance, it also regulates DV patterning through the induction of *En1*, which in turn restricts *Wnt7a* to the dorsal ectoderm [17], [21]–[23].

Sp6 and Sp8, also known as epiprofin and buttonhead, respectively, are two members of the Sp transcription factor family that have been implicated in AER induction and maintenance [24]–[27]. Both share similar patterns of expression in the limb bud ectoderm and AER and function downstream of Wnt/βcatenin signaling and upstream of *Fgf8*. Based on their overlapping patterns of expression and on their individual loss-of function phenotypes, we suspected that these two factors act in a complementary manner in the induction and maintenance of the AER downstream of Wnt/βcatenin [26]. Therefore, in order to further elucidate the functions and potential redundancy of these two genes, we generated double *Sp6*;*Sp8* null mutants. We also generated *Sp6-*null;*Sp8*-conditional mutants using an *Sp8* floxed allele with both the *AP2αCre* and the *Msx2Cre* deleter lines. Interestingly, mutant embryos that lacked the four *Sp6;Sp8* alleles or that retained a single *Sp6* allele were tetra-amelic. Initial budding occurred, but *Fgf8* was not activated in the limb ectoderm preventing further development. Mutants bearing a single functional copy of *Sp8* displayed a split-hand/foot malformation phenotype (SHFM) with dorsalization of the digital tips. The phenotypic data together with the molecular defects identified in mutant limb buds indicate that Sp6 and Sp8 are together absolutely necessary for AER development and DV patterning.

## Results

Both *Sp6* and *Sp8* are expressed in the entire prospective limb ectoderm and progressively become confined to the AER as the limb bud emerges. Loss of function of *Sp6* [26], [28] results in soft tissue syndactyly in the forelimb and osseous syndactyly to complete phalangeal synostosis in the hindlimb, whereas the inactivation of *Sp8* [24], [25] results in variable limb truncations most frequently at the level of the elbow/knee. Both mutations show a deficit in the maturation of the AER. Also, dorsalization of the ventral digit tips is a characteristic feature of *Sp6* mutants and the molecular analysis of *Sp8* mutants indicates that early limb buds become progressively dorsalized [24]–[26]. Although the individual inactivation of either *Sp6* or *Sp8* does not interfere with the initial activation of *Fgf8* in the AER, several studies have demonstrated that both factors function downstream of Wnt/βcatenin signaling and that Sp8 is able to bind and activate the *Fgf8* promoter [26], [27], [29], [30]. This, together with their similar expression patterns in the limb ectoderm, led us to propose that Sp6 and Sp8 transcription factors have a redundant function in the Wnt/βcatenin dependent induction of *Fgf8* in the AER [26].

We have previously shown that *Sp8* expression is maintained in the absence of *Sp6* [26] and here we found that *Sp6* is expressed in the absence of *Sp8* although at a lower level than normal, and is progressively downregulated in concert with the downregulation of *Fgf8* expression (Figure S1 and [24], [25]). Thus, *Sp6* may directly or indirectly require Sp8 to maintain a normal level of expression. Nevertheless, the expression of *Sp6* even at a reduced level in the *Sp8* mutant could account for the induction and partial maintenance of *Fgf8* supporting our hypothesis that both factors function in a redundant manner during limb development.

### Limb phenotype of double *Sp6;Sp8* mutants

**Figure 1:**
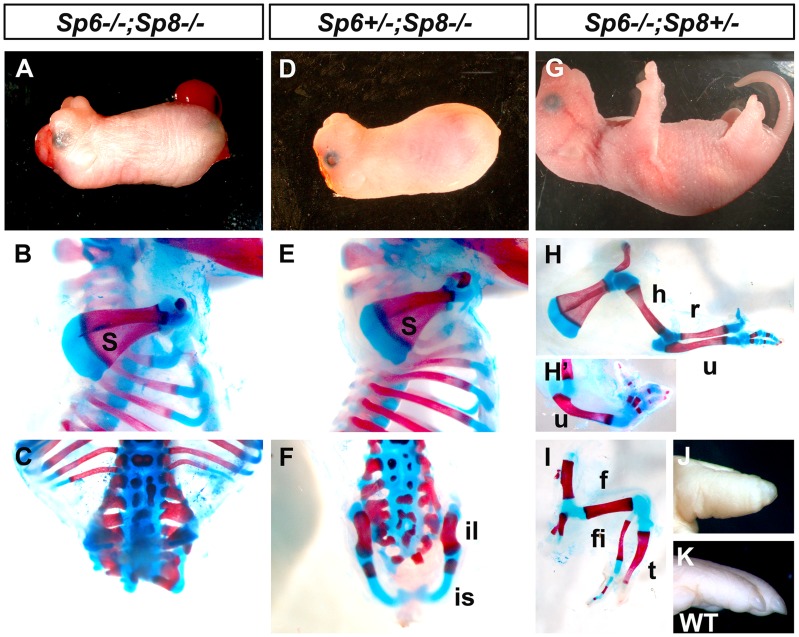
Effects of inactivating *Sp6* and *Sp8* in limb development. The external aspect (top row) and skeletal preparations of the forelimb (middle row) and hindlimb (bottom row) of newborns are shown for each genotype (genotypes indicated at the top). In the absence of the four functional alleles of *Sp6* and *Sp8* (A–C), or when only one functional allele of *Sp6* remains (D–F) no limbs form. Underdeveloped hip bones with rudimentary ilium and ischium form when one functional allele of *Sp6* is present (F). Animals with only one functional allele of *Sp8* (G–I) display a split hand/foot malformation phenotype with occasional absence of the radius (H′) and more severe phenotype in the hindlimb (I). The digit tips in these limbs show conical nails (J), compare with normal digits (K). Abbreviations: s, scapula; h, humerus; r, radius; u, ulna, f, femur, t, tibia, fi, fibula, is: ischium il, ilium.

To test our hypothesis we analyzed limb development in double *Sp6*;*Sp8* mutants (Figure 1). For this genetic approach, we used the *Sp6* (*Sp6^−^*; [28]) and the *Sp8* (*Sp8^CreERT2^*, hereafter referred to as *Sp8^−^*; [24]) null alleles and we analyzed the progeny from crosses between *Sp6^+/−^;Sp8^+/−^* double heterozygous mice (Figure 1). *Sp6^+/−^;Sp8^+/−^* double heterozygous mice showed no obvious defect in either limb patterning or skeletogenesis, yet displayed subnormal fertility. Skeletal preparations of the neonates recovered from these crosses were used to characterize the limb phenotype; other phenotypic traits will be considered elsewhere.

**Figure 2:**
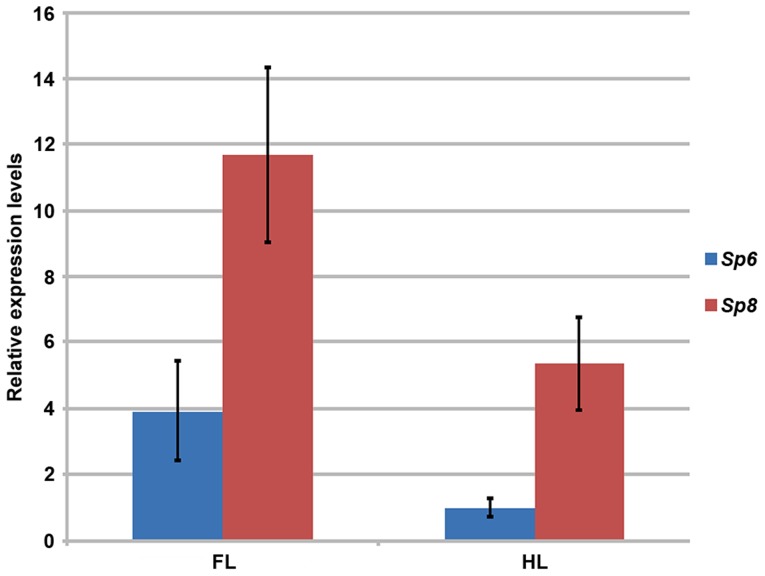
RT-qPCR quantification of *Sp6* and *Sp8* transcripts in the limb ectoderm of E10.5 control embryos. Histogram bars represent the average expression values after normalization to the ubiquitously expressed 18s-RNA (standard deviation shown as error bars). *Sp8* (red) exhibits a higher level of expression than *Sp6* (blue) both in forelimbs (FL) and in hindlimbs (HL) and both factors are expressed at higher level in the forelimb than in the hindlimb.

Animals singly mutant for *Sp6* or for *Sp8* exhibited their previously described phenotypes including exencephaly and spina bifida in *Sp8* mutants [24]–[26]. In our crosses, the majority of *Sp8* mutant limbs were truncated at the level of the elbow/knee with the olecranon also present in half of the specimens. Remarkably, in 100% of newborn double mutants both forelimbs and hindlimbs were absent (Figure 1A–C; 3 out of 102). In these mutants, no skeletal elements formed distal to the scapula (Figure 1B). Caudal lumbar vertebrae were highly disorganized and the body appeared truncated caudal to the sacrum with only rudimentary cartilage contributing to the pelvis (Figure 1C and Figure S2). Also, animals in which both copies of the *Sp8* gene and one copy of the *Sp6* gene had been removed (*Sp6^+/−^;Sp8^−/−^*) were always tetra-amelic (Figure 1D–F; 10 out of 102). However, in contrast to double mutants, the pelvic girdles showed undeveloped iliac and ischial anlagen (Figure 1F and Figure S2). The effect of a single functional copy of *Sp6* in the morphogenesis of the pelvic girdle is shown in detail in Figure 2.

Mutant mice in which both copies of the *Sp6* gene and one copy of the *Sp8* gene had been inactivated (*Sp6^−/−^;Sp8^+/−^*) had proximal-distal (PD) complete, but extremely malformed, limbs (Figure 1G–I, 11 out of 102). Consistently, the forelimb paw had the “claw-like” appearance typical of split-hand/foot malformation (SHFM) in which anterior digits were hypoplastic or missing and posterior digits were frequently fused (Figure 1G–H–H′). The radius was occasionally absent (Figure 1H′). Hindlimbs showed a more severe phenotype with the zeugopod constantly abnormal (Figure 1I). Although there was some variability, the majority of specimens displayed a misshaped and frequently truncated tibia and a thin fibula surmounted by one or two rows of small skeletal rods that we interpreted as digits (Figure 1I). The phenotype was variable among different animals and within individuals each paw showing specific deficiencies. No left or right severity preference was identified. This phenotype is comparable to the human SHFM, a highly variable malformation that has also been termed ectrodactyly, split hand, cleft hand and lobster claw hand [31]–[34]. Of most interest, the digits in both fore and hindlimbs of *Sp6^−/−^;Sp8^+/−^* mutants were bidorsal exhibiting circumferential nails and lacking ventral pads (Figure 1J–K).

In summary, our genetic analysis shows that Sp6/Sp8 transcription factors are together absolutely required for limb development. Furthermore, the data support our hypothesis that Sp6 and Sp8 perform complementary functions in the limb ectoderm. Interestingly, one single functional allele of *Sp6* is insufficient, in the absence of an *Sp8* allele, to support limb development. In contrast, one single functional allele of *Sp8*, in the absence of an *Sp6* allele, permits development of all three PD segments, although displaying a SHFM phenotype.

### *Sp8* is expressed at higher level than *Sp6* in the limb ectoderm

Since *Sp6* and *Sp8* display similar temporal and spatial patterns of expression in the limb ectoderm [24]–[26], one possible explanation for the difference in their functional capacity as described above is that *Sp8* has specific functions that *Sp6* cannot accomplish. However, it is also possible that these functional differences are due to differences in their levels of expression. Thus, to quantify the *Sp8* and *Sp6* levels of expression in the limb ectoderm we performed a quantitative RT-PCR assay in E10.5 control embryos. Our results showed that *Sp8* was expressed more robustly than *Sp6* during limb development (Figure 2). Expression of *Sp8* was 3 fold higher than expression of *Sp6* in the forelimb and 5 fold higher in the hindlimb. Our quantitative analysis also showed that the expression of both *Sp6* and *Sp8* was higher in the forelimb than in the hindlimb, although it should be noted that the development of the hindlimb is delayed compared to that of the forelimb at this stage, which could account for the forelimb/hindlimb disparity.

To investigate the basis for the differential level of expression of *Sp6* and *Sp8* in the limb, we performed an *in silico* analysis of their putative promoter regions (Figure S3). To enhance the identification of functionally relevant regulatory sequences, we limited our evaluation to regions 5′ of the coding sequences that were conserved across divergent species as determined by the mVista browser [35]. We further screened the conserved regions for potential transcription factor binding sites using Alibaba 2.1 and Sequencher 4.8 and then confirmed conservation between mouse and human. Our analysis identified 12 potential βcatenin/Lef1 binding sites 5′ to the *Sp8* coding sequence, whereas *Sp6* had only five. This finding provides a potential mechanism for the increased level of *Sp8* transcription during limb development. In addition, the presence of 29 potential Sp binding sites in the region containing the putative *Sp6* promoter and the 12 present in *Sp8* supports a possible cross-regulation between Sp transcription factors as suggested by the lower *Sp6* expression in absence of Sp8 (Figure S1). Based on our quantitative and *in silico* analysis we speculate that Sp8 makes a more substantial contribution to limb development than Sp6 because of a higher level of transcription, a speculation that requires further investigation.

### *Ap2αCre* inactivation of *Sp8* on an *Sp6* deficient background

When performing the crosses between double heterozygous we found a reduced frequency of pregnancies in double heterozygous females and also that the fraction of double mutant offspring was significantly below the expected 1/16 Mendelian frequency. To circumvent these issues and avoid the neural phenotype from *Sp8* null mutants, we used an *Sp8* floxed conditional allele (*Sp8^f^*; [36]) to remove it specifically from the limb ectoderm. Among the available lines with Cre activity in the limb ectoderm (*Msx2Cre* [37]; *Brn4Cre* [17]; *RARCre* [38]; *AP2αCre* [39]; *Mox2Cre* [40]), we selected the *AP2αCre* line because it has been reported to drive very early Cre function in both fore and hindlimbs, at least before activation of *Fgf8* [41]. Because *Sp8* is already expressed at E7.5 in the embryonic ectoderm ([24], [25] and authors' personal observations), we decided to determine in more detail the activity of the *AP2α;Cre* transgenic line using the ROSA26 reporter strain (R26R; [42]). Our analysis showed *AP2α;Cre* activity in the early embryonic ectoderm at E8.5 indicating that the removal of the *Sp8* floxed allele would occur before limb initiation (Figure S4).

Thus, we used the *AP2α;Cre* line, the conditional allele of *Sp8* and the *Sp6* null allele to generate the combined loss of function of *Sp6* and *Sp8* in the limb ectoderm (Figure S5). As to be expected, the double mutants (*Sp6^−/−^;Sp8^f/−^;AP2αCre*) and the mutants that retained a single allele of *Sp6* (*Sp6^+/−^;Sp8^f/−^;AP2αCre*) were 100% tetra-amelic and showed similar phenotypes to those described above for the double ubiquitous deletions (Figure S5A–F). Also as expected, the *Sp6^−/−^;Sp8^f/+^;AP2αCre* genotype exhibited the SHFM phenotype with its typical variability (compare Figure 1H–I with Figure S5H–I). In sum, the limb phenotypes obtained using the *Sp8* floxed allele and the *AP2αCre* line replicated exactly the phenotypes obtained with the constitutive deletions. Finally, it should be noted that the neural phenotype was not rescued in conditional mutants (Figure S5A, 5D) indicating an unanticipated wide overlap between the expression of *AP2α* and *Sp8* in the neural tube (Figure S4).

### *Msx2Cre* inactivation of *Sp8* on an *Sp6* deficient background

We also inactivated *Sp8* from the limb ectoderm using the *Msx2;Cre* line simultaneously with the inactivation of *Sp6*. This *Msx2;Cre* transgenic line has been extensively monitored using the ROSA26 reporter strain [15], [37] and it is known that it drives Cre activity before *Fgf8* activation of expression in the hindlimb but after *Fgf8* expression and initiation of limb development in the forelimb. We reasoned that the use of this conditional mutant would provide information on the requirement of *Sp8* once the early stages of limb initiation have occurred.

**Figure 3:**
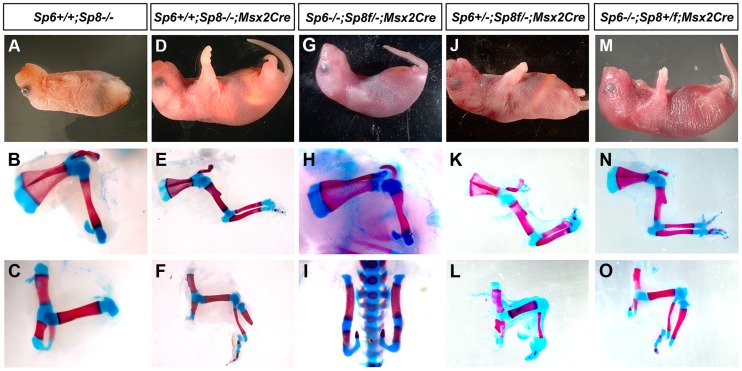
*Msx2Cre* removal of *Sp8* on a *Sp6* deficient background. The external aspect (top row) and skeletal preparations of the forelimb (middle row) and hindlimb (bottom row) of newborns are shown for each genotype (genotypes indicated at the top). *Msx2Cre* conditional removal allows transient expression of *Sp8* in both forelimbs and hindlimbs which results in *Sp8* conditional mutant (D–F) displaying a milder limb phenotype than ubiquitous mutants (A–C). One single conditional allele of *Sp8* in the forelimb (G–H) seems to be equivalent to both functional alleles of *Sp6* (A–B) while in the hindlimb is not sufficient for limb development (C, I). This conditional allele of *Sp8* in addition to one single allele of *Sp6* permits the formation of the three PD segments of the limb although with a single digit (J–L). Finally, one conditional allele of *Sp8* plus a normal allele *Sp8* results in SHFM (M–O).

First of all we compared the phenotype of the limb conditional *Sp8* mutant (*Sp8^f/−^;Msx2Cre*) with that of the *Sp8* null mutant (*Sp8^−/−^*) in both forelimbs and hindlimbs (Figure 3A–F). Notwithstanding the variability, the phenotypes using the conditional allele were on average milder than those using the constitutive null allele [24], [25], [30] (Figure 3A–F). In the conditional *Sp8^f/−^;Msx2Cre* mutant, an initial burst of *Sp8* expression permitted normal forelimb development up to the wrist and furthermore one or two incomplete posterior digits were formed (Figure 3E). In the hindlimbs, one posterior digit was always present although the tibia frequently appeared truncated (Figure 3F). This improvement in the phenotype (compare Figure 3A–C with Figure 3D–F) indicates that a transient early expression of *Sp8* has a considerable impact on both fore and hind limb development.

The conditional removal of *Sp8* in the absence of *Sp6* (*Sp6^−/−^;Sp8^f/−^;Msx2Cre*) resulted in a forelimb truncated at the elbow while the hindlimbs didn't develop (Figure 3G–I). When one copy of *Sp6* remained (*Sp6^+/−^;Sp8^f/−^;Msx2Cre*) the phenotype notably improved with truncations at the level of the wrist/ankle associated with the formation of an incomplete digit (Figure 3K–L). Finally, when a functional copy of *Sp8* remained besides the *Sp8* floxed allele (*Sp6^−/−^;Sp8^+/f^;Msx2Cre*) the phenotype obtained was SHFM (Figure 3M–O).

In summary, when the phenotypes of our allelic series are classified according to severity, a clear correlation with the total dosage of *Sp6* and *Sp8* is observed (Figure 1, 3 and Figure S5). The more parsimonious explanation is that both transcription factors are functionally equivalent during limb development, although Sp8 makes a greater contribution than Sp6 presumably due to a higher level of expression (Figure 2). Our study also suggests that there is a threshold of expression below which no limb forms and that the level of *Sp6* expression attained by a single allele of *Sp6* is below this threshold.

### A functional AER does not develop when the gene dosage of *Sp6* and *Sp8* is significantly reduced or completely eliminated

**Figure 4:**
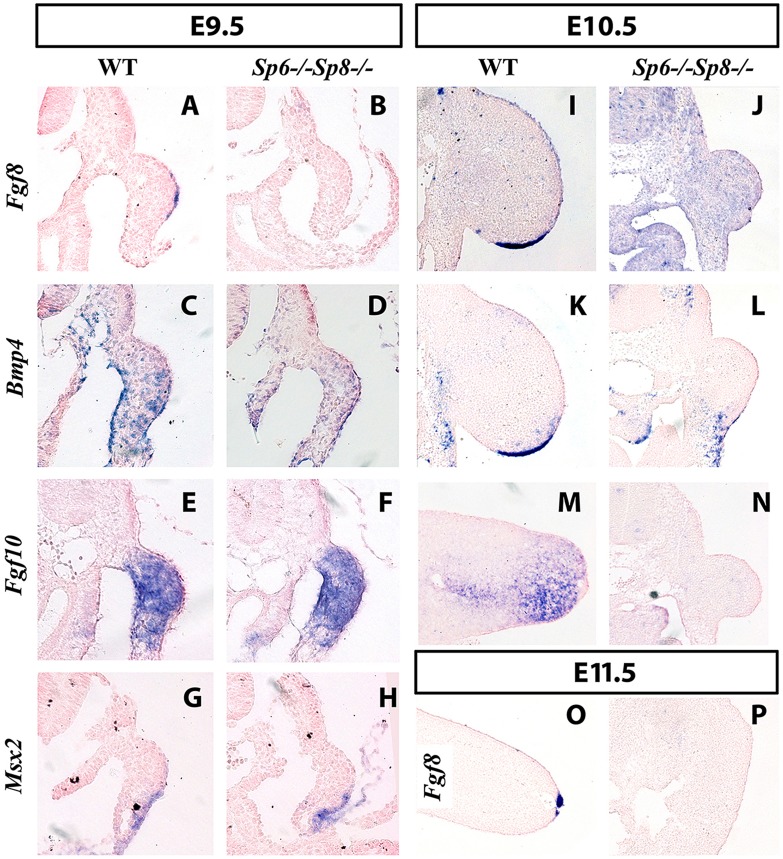
*Fgf8* is not detected in double *Sp6;Sp8* mutants. ISH to transverse sections through the level of the forelimbs at the stage indicated at the top and with the probe indicated on the left. Genotypes are also marked at the top of the figure. In the absence of *Sp6* and *Sp8*, *Fgf8* expression in the limb ectoderm is never detected as shown at E9.5 (A–B), E10.5 (I–J) and E11.5 (O–P). However, *Bmp4* (C–D), *Fgf10* (E–F) and *Msx2* (G–H) are normally activated at E9.5 but not maintained at later stages (K–N). Note that the initial budding of the double mutant is similar to normal (A–H) but further growth is impaired (I–N) and complete regression has occurred by E11.5 (O–P). In all panels dorsal is up and distal to the right.

Since both Sp6 and Sp8 are involved in the Wnt/βcatenin dependent induction of *Fgf8*, it seems reasonable to presume that the amelic phenotype of double mutants may rely on a failure to induce a functional AER. Therefore we examined embryonic limbs at the stages when the limb bud is emerging and the AER is being induced. For this analysis we used the *Sp6* and *Sp8* constitutive null alleles. By E9.5, in the normal limb bud, several genes including *Fgf8*, *Bmp4* and *Msx2* are expressed in the ventral limb ectoderm forming the preAER [17], [22], [43]. These AER precursors will become progressively confined to the distal tip as the AER matures [7], [20] (Figure 4A, C, G, I, K, O).

However, in the absence of the four *Sp6;Sp8* alleles (*Sp6^−/−^;Sp8^−/−^*) or when only one functional allele of *Sp6* remained (*Sp6^+/−^; Sp8^−/−^*), *Fgf8* was never detected in the limb ectoderm at any of the stages analyzed (Figure 4B for E9.5 (25–30 somites); Figure 4J for E10.5 (36–40 somites) and Figure 4P for E11.5). Because these two genotypes always showed identical expression patterns for all the genes analyzed, only the results of *Sp6^−/−^;Sp8^−/−^* mutants are shown in the Figures. In contrast to *Fgf8*, *Bmp4* and *Bmp2* expression was found to occur normally at E9.5 both in the limb ectoderm and limb mesoderm of double mutants and mutants with a single functional allele of *Sp6* (Figure 4C–D). This was confirmed by the expression of *Msx2*, a *bona fide* target of Bmp signaling [44], [45] (Figure 4G–H). However, neither *Bmp4* (Figure 4K–L) nor *Msx2* were maintained in the limb ectoderm by E10.5. Disregarding the absence of *Fgf8* expression, initiation of limb development was normal in *Sp6^−/−^;Sp8^−/−^* and *Sp6^+/−^;Sp8^−/−^* compound mutants with the formation of a small bulge; thus by E9.5 the phenotype was not yet evident (Figure 4A–H).

The current view considers that Fgf10 signaling from the limb mesoderm induces Wnt/βcatenin signaling in the ectoderm and this leads to *Fgf8* activation and therefore AER induction in the ectoderm. Subsequently, Fgf8 from the ectoderm signals back to the mesoderm to maintain Fgf10, establishing an Fgf10-Fgf8 positive feedback loop necessary for further outgrowth [11]. Consistent with Sp6 and Sp8 acting downstream of *Fgf10* and Wnt/βcatenin signaling, double mutant limb buds normally activated *Fgf10* expression in the limb mesenchyme (Figure 4E–F at E9.5). However, due to the failure to activate *Fgf8*, the emergent limb buds cannot maintain *Fgf10* in the limb mesoderm (Figure 4M–N) and regress so that by E11.5 no trace of the limb bud remained (Figure 4O–P).

These results demonstrate the absolute requirement of Sp6/Sp8 for *Fgf8* activation in the limb ectoderm and are consistent with Sp6/Sp8 being necessary mediators of Wnt/βcatenin induction of *Fgf8*. Finally, our results also show that *Bmp4* expression in the limb ectoderm, which requires βcatenin [14], [15], can occur in the total absence of Sp6 and Sp8 (this work, see Figure 4C–D) as well as in the absence of significant AER-related *Fgf* expression [46].

### In the absence or significant reduction of *Sp6/Sp8*, limb development initiates, but later regresses by apoptosis

Next we investigated the reason of the regression of the emerging limb bud in double mutants. The phenotype of the double mutants is reminiscent of the chick mutant *limbless*. In *limbless* the limb bud arises normally, but due to the inability to form an AER, the entire bud undergoes cell death and disappears [47], [48]. Also, cell death is a constant feature after the surgical removal of the AER [3], [4] or genetic attenuation of Fgf signaling from the AER [38], [46], [49], [50]. Therefore, we analyzed cell death by TUNEL in our double mutant limb buds.

**Figure 5:**
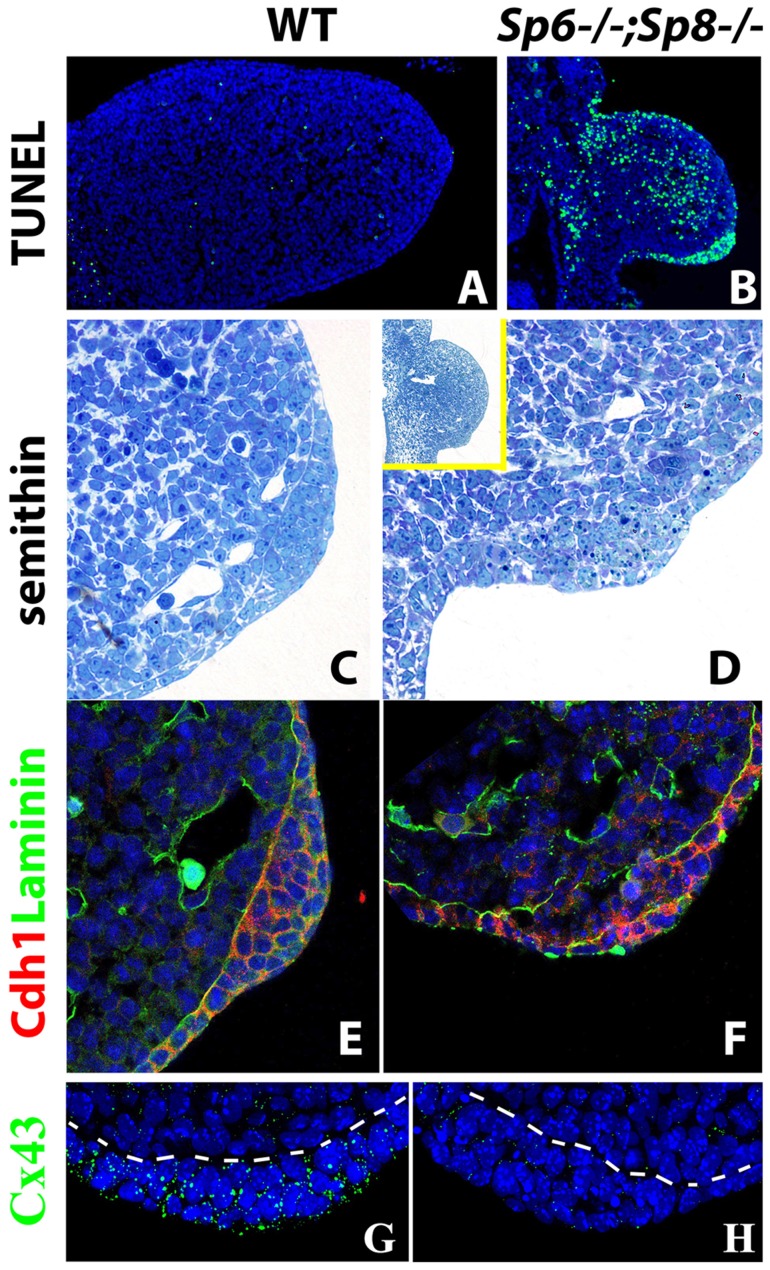
Effects of inactivating *Sp6* and *Sp8* genes on cell survival and AER morphogenesis. (A–B) TUNEL assay showing abundant apoptotic cells (green) both in the mesoderm and ectoderm of the E10.5 double mutant forelimb bud (B) compared to control (A). (C–D) semithin longitudinal section of araldite embedded control and double mutant limb buds showing the thickening in the ventral ectoderm of mutants. The insert in D shows a lower magnification to appreciate the ventral position of the ectoderm thickening in mutants. (E–F) Confocal images of double immunohistochemistry for Laminin-b, marking the basement membrane (green) and E-cadherin expressed specifically in the ectoderm (red) showing that the cells accumulated in the ventral limb ectoderm of mutant embryos are of ectodermal origin. (G–H) Confocal images of Connexin 43 immunostaining showing the enrichment of gap junctions in the control AER (green dots) but not in the double mutant AER. All the panels show forelimb buds at E10.5. In the immunostainings, the nuclei are counter stained with DAPI (blue).

Abnormal cell death, compared with control littermates, was not detected at E9.5 in *Sp6^−/−^;Sp8^−/−^* and *Sp6^+/−^;Sp8^−/−^* compound mutants. However, extensive apoptosis was apparent both in the mesoderm and ectoderm of these mutant limb buds by E10.5 (Figure 5A–B). Cell death started and was most prominent in the central region of the bud but apoptotic cells were also observed in the ectoderm particularly at dorsal proximal and ventral level (Figure 5B). This extensive apoptosis can account for the regression of the limb bud and the amelic phenotype as in *limbless* [47], [48].

### AER morphogenesis initiates even in the complete absence or significant reduction of *Sp6* and *Sp8*

In the histological sections of double mutant limb buds we noticed a thickening of the ventral ectoderm that was particularly evident in the TUNEL assays because of the abundant cell death in this region (Figure 5A–B). To analyze this thickening with maximum detail, we performed semithin sections (1 micron thick) of araldite embedded embryos. Transverse sections through double mutant (*Sp6^−/−^;Sp8^−/−^*) and *Sp6^+/−^;Sp8^−/−^* embryos at the level of the forelimbs showed an irregular thickening of the ventral ectoderm by E10.5 (Figure 5C–D). The thickening didn't span the whole ventral ectoderm but was patchy and sometimes protruded into the mesoderm; it had the appearance of a ventrally positioned and immature AER, in which the apoptotic images were very abundant. To confirm that this thickening was of ectoderm origin, we used immunohistochemistry and confocal microscopy to localize E-Cadherin (Cdh1), which is an epithelial marker, and laminin, a major component of the basement membrane. The double immunohistochemistry demonstrated that the thickening was ectodermal as it expressed Cdh1 and was underlined by a laminin marked basement membrane (Figure 5E–F). To assess the functionality of this thickened ectoderm, we analyzed the expression of Connexin 43 (Cx43), a gap junction protein encoded by the *Gja1* gene and considered a marker of the specialized AER ectoderm [51]. In contrast to the high expression present in the wild type AER, Cx43 was not detected above background in the thickened ectoderm of double mutants (Figure 5G–H).

Taken together our results indicate that Sp6/Sp8 factors are absolutely required for a functional AER, but dispensable for initial AER morphology confirming an independence between AER morphology and function.

### Absence or significant reduction of Sp6/Sp8 activity in the limb ectoderm disrupts DV patterning

The known relationship between the specification of the AER and DV patterning together with the DV phenotypic alterations present in *Sp6^−/−^* and *Sp6^−/−^;Sp8^+/−^* mutants prompted us to analyze the state of DV patterning in our mutants. Furthermore, the ventral position of the mutant AER indicates a failure in the normal morphogenetic movements of the ectoderm that compact the AER, a process in which *En1* has been implicated [6], [20]. Thus, we analyzed the expression of two genes relevant to DV patterning, *Wnt7a* and *En1*, in consecutive serial limb bud sections.

**Figure 6:**
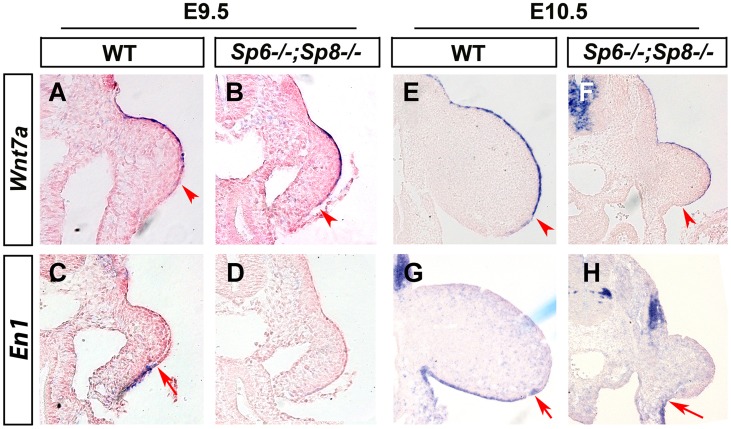
Effects of inactivating *Sp6* and *Sp8* genes on dorsal-ventral limb patterning. ISH to transverse sections through the level of the forelimbs at the stage indicated at the top and with the probe indicated on the left. Genotypes are also marked at the top. Note that, contrary to controls (A, E), *Wnt7a* is not restricted to the dorsal ectoderm in double mutant embryos (B, F). Accordingly, *En1* expression is undetectable in the ventral limb ectoderm of mutant embryos (D, H) compared to controls (C, G). The arrowheads and arrows mark the distal limit of *Wnt7a* and *En1* expression, respectively.

In the emerging limb bud (E9.25; 22–23 So), before *En1* expression is detectable, *Wnt7a* is normally expressed in the dorsal ectoderm exceeding the mid-distal point of the bud and extending into the ventral ectoderm [7], [23] (author's personal observations). Shortly afterwards, the expression of the pre-AER markers *Fgf8* and *Bmp4* in the ventral ectoderm and of *En1* in the more proximal ventral ectoderm progressively restricts *Wnt7a* to the dorsal ectoderm (Figure 6A, C).

In double mutant *Sp6^−/−^; Sp8^−/−^* and *Sp6^+/−^;Sp8^−/−^* embryos, the initial extended expression of *Wnt7a* was never restricted to the dorsal ectoderm and its expression persisted covering almost the entire limb ectoderm while *En1* expression was not detected in the ventral ectoderm (Figure 6B–D, F–H). These results reveal that the absence or significant reduction of *Sp6*/*Sp8* dosage interferes with the normal specification of DV patterning resulting in double dorsal distal limb buds. Our results also show that a virtually normal Bmp signaling in the early limb bud (Figure 4C–D and Figure 4G–H) is not sufficient for *En1* expression in the absence of *Sp6* and *Sp8*.

### Mutants retaining a single functional allele of *Sp8* exhibit a split-hand/foot malformation

The presence of a single allele of *Sp8* (*Sp6^−/−^;Sp8^+/−^* or *Sp6^−/−^;Sp8^f/+^*;*AP2αCre* or *Sp6^−/−^;Sp8^f/+^*;*Msx2Cre*), was sufficient to allow the elaboration of all three segments along the PD axis, although the autopod was characterized by the loss or malformation of central elements creating a SHFM.

**Figure 7:**
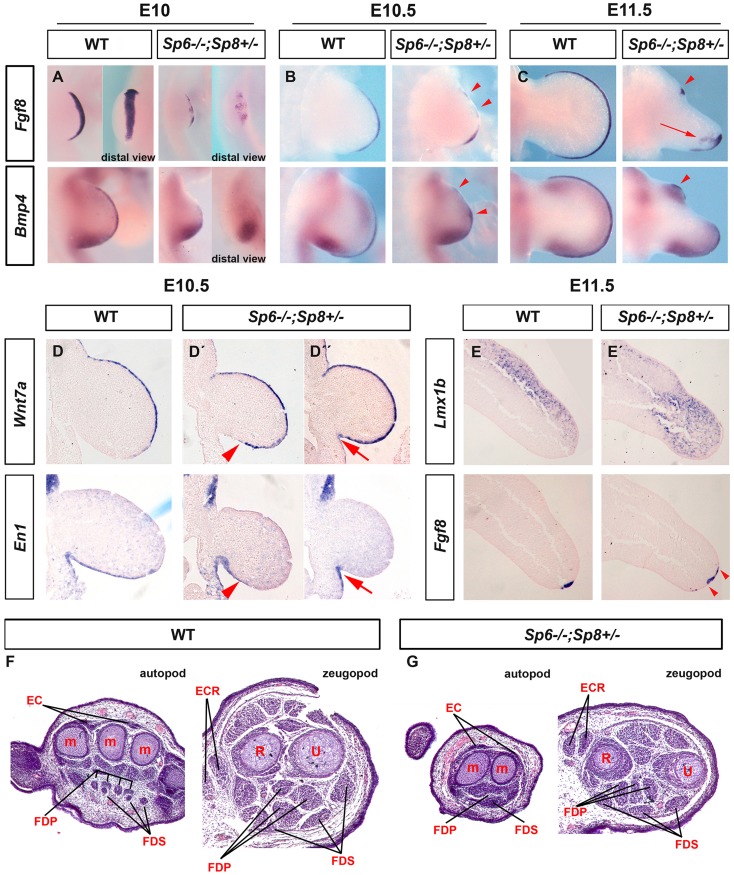
Molecular and morphological analysis of *Sp6−/−;Sp8+/−* mutant limb buds. (**A–C**) WMISH for *Fgf8* and *Bmp4* showing irregular activation in the limb bud ectoderm of *Sp6^−/−^;Sp8^+/−^* E10 (A), E10.5 (B) and E11.5 (C) forelimb buds compared to control littermates. Note the irregular early activation and predominant posterior maintenance of *Fgf8* and *Bmp4* expression, except for a residual focus of anterior expression (red arrowheads). (**D, D′, D″**) ISH for *Wnt7a* and *En1* to consecutive (7 microns apart) sections of control and mutant E10.5 forelimb buds (D, D′ and D″). Note the variable expansion of *Wnt7a* into the ventral ectoderm always associated with a corresponding proximal restriction of *En1* (D′, D″) indicated by red arrowheads (D′) and red arrows (D″). (**E–E′**) ISH for *Lmx1b* and *Fgf8* in consecutive sections of control and *Sp6−/−;Sp8+/−* E11.5 forelimb buds. The *Lmx1b* expression invades the ventral mesoderm distally under the broad and flat AER. (**F–G**) Hematoxylin-Eosin stained transverse histological sections at the autopod and zeugopod level of E15.5 control (F) and *Sp6−/−;Sp8+/−* (G) limbs. Some of the individual muscles and tendons are labeled. Abbreviations: EC, extensor digitorium communis; FDS, flexor digitorium sublimis; FDP, Flexor digitorium Profundus; ECR, extensor carpi radiallis; m, metacarpal; R, radius; U, ulna.

To understand the molecular basis of this phenotype, we analyzed the expression of *Fgf8* during limb development in *Sp6^−/−^;Sp8^+/−^* mutants. This analysis showed that the AER precursors were irregularly specified in the ventral ectoderm. The whole mount in situ hybridization at E10 showed obvious gaps and irregularities in the area in which *Fgf8* should be uniformly expressed (Figure 7A). During further development, the expression of *Fgf8* became robust in the posterior AER, but was absent in the central-anterior areas except for a typical spot of residual anterior expression (Figure 7B–C). The expression of *Bmp4* in the ectoderm always replicated the same abnormal pattern as *Fgf8* (Figure 7A–C). Furthermore, the compaction and maturation of the AER was defective as it remained flat and broad with occasional extensions into the ventral ectoderm (arrow in Figure 7C and 7E′). Thus, in harmony with previous reports [32], [52], [53], the SHFM phenotype in our *Sp6^−/−^;Sp8^+/−^* mutants derives from a failure to properly establish and maintain the AER, preferentially in the central to anterior limb region.

In *Sp6^−/−^;Sp8^+/−^* mutants the expression of *Wnt7a* and *En1* was consistently abnormal but highly variable even within a single limb bud. *Wnt7a* was always found to abnormally extend into the ventral ectoderm to a variable degree that always correlated with a complementary ventral expression of *En1* (Figure 7D, D′, D″). This was easily appreciated when consecutive sections of the same limb bud were hybridized for *Wnt7a* and *En1* as shown in Figure 7D′ and 7D″. Accordingly, the expression of *Lmx1b*, the downstream target of Wnt7a responsible for the dorsalization of the dorsal mesoderm [54], was found to variably extend under the flattened and broad AER into the ventral mesoderm distally (Fig. 7E–E′). These molecular alterations explain the bidorsal tips of *Sp6^−/−^;Sp8^+/−^* mutants. To ascertain possible DV defects at more proximal levels we performed a histological analysis on transversal sections of E15.5 mutant and control limbs. Our results showed that DV patterning of muscles and tendons were preserved for the most part in the stylopod and zeugopod, but were less well defined in the autopod (Figure 7F–G).

**Figure 8:**
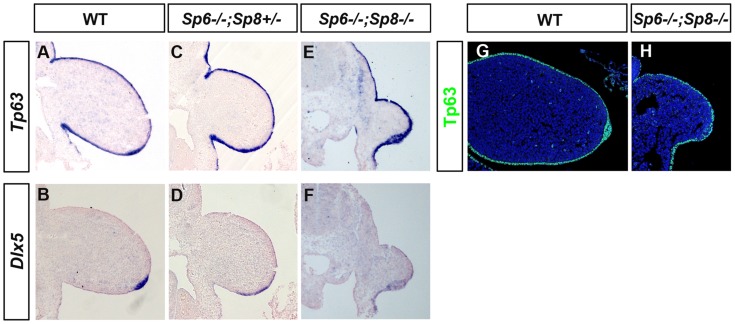
*Tp63* and *Dlx5* expression in mutant limb buds. (**A–F**) *Tp63* and *Dlx5* expression is normally detected in the limb ectoderm of control (A, B), *Sp6−/−;Sp8+/−* (C, D) and *Sp6−/−;Sp8−/−* (E, F) mutants although *Dlx5* is downregulated. (**G–H**) Immunostaining for Tp63 (green) showing expression in the *Sp6^−/−^;Sp8^−/−^* double mutant limb bud similar to wild type littermate. All the panels show longitudinal sections of E10.5 forelimb buds.

In humans, isolated or non-syndromic SHFM is a genetically heterogeneous developmental disorder of which six loci have been identified [31]–[34]. SHFM Type I, the most frequent variety, is due to a mutation on chromosome 7, in a region that contains the two homeobox genes *DLX5* and *DLX6* [55]–[57]. SHFM Type IV maps to chromosome 3 and it has been shown that *TP63* is the gene involved [31], [58]. Furthermore, it has been shown that *Dlx5* and *Dlx6* are transcriptional targets of Tp63 [59], [60]. Tp63 is a member of the p53 family of transcription factors crucial for stratified epithelial differentiation [61], [62] and Dlx5 and Dlx6 are members of the family of distalless-related homeodomain transcription factors (Dlx1–Dlx6) that play key roles in limb development. Therefore, we analyzed the expression of *Tp63* and *Dlx5* and *Dlx6* in our SHFM mutants, to determine whether the Tp63 pathway was involved. Our analysis showed that *Tp63* and *Dlx5* and *Dlx6* were normally expressed in the *Sp6^−/−^;Sp8^+/−^* mutant except for the flattened AER morphology (Figure 8A–D). Finally, the analysis of double mutants showed that *Tp63*, *Dlx5* and *Dlx6* were initially expressed normally in the complete absence of *Sp6* and *Sp8* (Figure 8E–F) suggesting that if Sp6/Sp8 are components of the Tp63 network, they act downstream of Dlx5 and Dlx6. The expression of Tp63 in *Sp6^−/−^;Sp8^−/−^* mutants was further confirmed by immunohistochemistry (Figure 8G–H).

## Discussion

### *Sp6* and *Sp8* play complimentary functions in limb development

There are numerous examples in limb development of related genes with similar patterns of expression playing redundant functions and therefore providing robustness to the system. Among these are members of the Fgf, Bmp, and Hox gene families in which the overall final gene dosage is the key parameter for normal morphology [49], [63], [64]. Here, by using a variety of loss-of-function alleles we have identified that *Sp6* and *Sp8* control AER development and DV patterning in a redundant and dose-dependent manner. However, both genes do not contribute equally which may in part be due to their differential levels of transcription.

**Figure 9:**
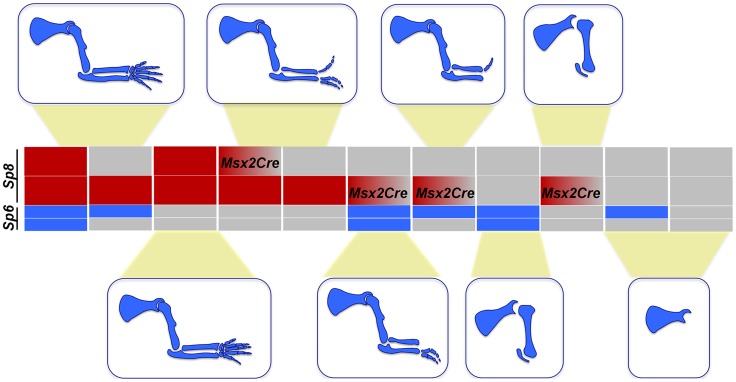
Illustration showing the correlation between the *Sp6/Sp8* gene dose and the severity of the limb phenotype. Blue boxes represent the *Sp6* alelles and red boxes the *Sp8* alelles. Grey boxes represent null alleles and boxes with a red to grey graduation represent conditionally removal with the *Msx2Cre* allele.

Notwithstanding the phenotypic variation associated with each particular genotype, when the predominant phenotypes obtained from the allelic series of compound *Sp6* and *Sp8* mutants are categorized in order of increasing severity, a strong correlation with gene dosage is observed (schematically shown for the forelimb phenotypes in Figure 9). A progressive reduction in the dose of *Sp6* and *Sp8* gene products leads to predictable morphology, from syndactyly, to SHFM, oligodactyly, truncation and finally amelia. This comparative analysis shows that the amount of gene product provided by a single functional allele of *Sp8* permits the complete development of the PD axis while one functional allele of *Sp6* does not, most likely because the gene product provided is below the critical threshold required for AER induction. Both alleles of *Sp6* provide less gene product than a single allele of *Sp8* and equivalent to a transient expression of one copy of *Sp8*, as occurs in the forelimb when the *Msx2;Cre* deleter line is used. Collectively, the data from our allelic series indicate that *Sp6* and *Sp8* are, for the most part, functionally equivalent and work in concert during limb development.

We found that the putative *Sp8* promoter has an increased number of potential βcatenin/Lef1 binding sites compared to *Sp6*, which might account for the higher levels of *Sp8* expression. Interestingly, another member of the Sp family also expressed in the limb ectoderm, Sp9 [27], is unable to promote limb development in the absence of Sp6/Sp8 possibly because of its low level of expression [27]. Supporting this notion, there is a decreased number of βcatenin/Lef1 binding sites within the *Sp9* putative promoter region when compared to *Sp6*.

We also considered whether the differences in Sp functional capacity could be due to structural differences. Comparative analysis of known protein domains and multiple alignment of Sp6, Sp8 and Sp9 revealed variability in the amino ends with the only common domains shared by these transcription factors being the zinc finger domains located in the carboxy ends. No structure-function correlation in the variable amino terminal domains was evident. For example, Sp6 and Sp8 are structurally disparate, but function in a complementary fashion. In contrast, Sp9 is structurally more similar to Sp8, than Sp6 is to Sp8 and yet does not show a complementary function in the limb. Therefore, even though these factors differ in their amino terminal domains, which may be functional in a different context [65], it is reasonable to speculate that in the limb, their functional capacity relies on their level of expression; this remains to be demonstrated.

### *Sp6* and *Sp8* are absolutely necessary for *Fgf8* induction and maintenance

Two of the main phenotypic features in our allelic series are truncations and SHFM. Studies in different mouse models and experimental manipulations in chick have established that these phenotypes can result from perturbations in AER functioning [1] and, accordingly, our analysis showed that *Sp6/Sp8* are required for the formation and maintenance of a functional AER.

The first phase in the formation of the AER is the induction of AER precursor cells in the limb ectoderm characterized by the expression of *Fgf8*. This depends on at least three important signaling inputs: i) Fgf10 produced in the limb mesoderm and signaling through the Fgf receptor 2b (Fgfr2b) expressed within the ectoderm [12], [13], [66]–[70], ii) Wnt/βcatenin signaling produced in the limb ectoderm and signaling preferentially to the ventral limb ectoderm [14], [15] and iii) Bmp signaling, mainly from the limb ectoderm, but also possibly from the limb mesoderm that signals through the Bmpr1a receptor in the limb ectoderm [16], [17], [22], [71], [72]. Although the crosstalk between these three inputs is complex and not completely understood, both the Fgf10 and the Bmp signaling pathways have been shown to act upstream of Wnt/βcatenin signaling in the induction of the AER [14], [16].

**Figure 10:**
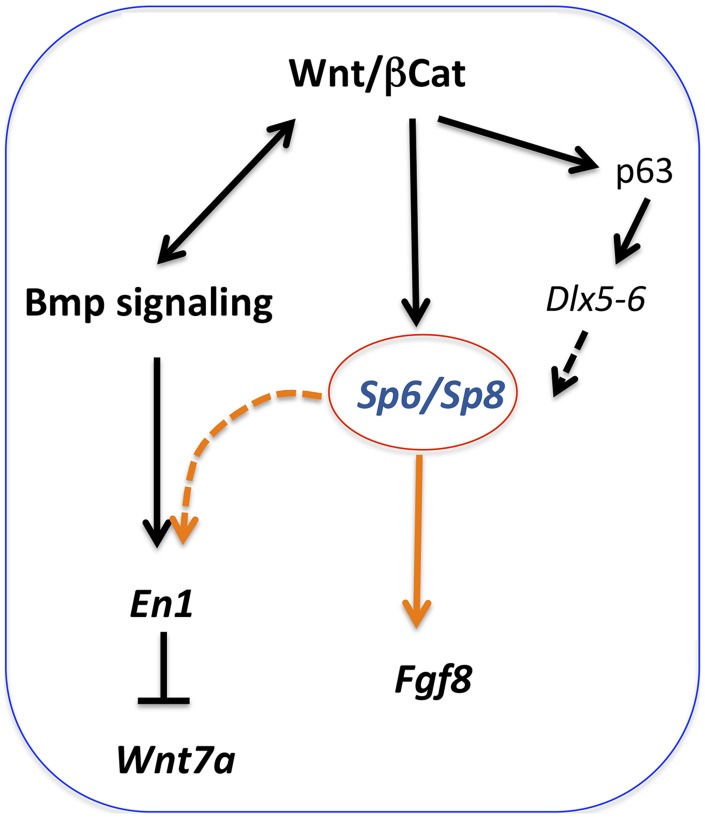
Regulatory pathways mediated by Sp6 and Sp8. *Sp6* and *Sp8* are necessary mediators of the Wnt/βcatenin-dependent induction of *Fgf8* in the limb ectoderm. In addition, these two factors also collaborate with BMP signaling in the induction of *En1* in the ventral limb ectoderm. Finally, Sp6 and Sp8 may also act downstream of *Tp63* and *Dlx* genes.

The analysis we have performed shows that when the dose of *Sp6/Sp8* is significantly reduced, *Fgf8* is not activated, disregarding initial normal *Fgf10* expression and Bmp signaling. Because both Sp6 and Sp8 have been shown to function downstream of Wnt/βcatenin signaling [26], [27], [30], and Sp8 has been shown to bind and activate the *Fgf8* promoter [29], our results fit with a model in which Sp6 and Sp8 function as transcriptional activators of *Fgf8* downstream of Wnt/βcatenin signaling in the limb ectoderm (Figure 10). Sp6 and Sp8 function together and in a dose-dependent manner as necessary mediators of the Wnt/βcatenin-*Fgf8* regulatory loop. Our phenotypic and molecular studies indicate that the level of gene product produced by a single *Sp8* allele is around the minimum dose required for the activation and maintenance of *Fgf8* expression while that produced by a single *Sp6* allele does not reach this minimum.

It is known that the Wnt/βcatenin signaling pathway is not only required for AER induction, but also for its maintenance. The limb truncations observed when, in the absence of *Sp6*, *Sp8* is removed from the forelimb ectoderm after the AER has been induced (*Sp6^−/−^;Sp8^f/−^;Msx2Cre*), indicate an ongoing role for Sp8 in AER maintenance, further supporting our model.

Most interestingly, our analysis shows that the complete absence of Sp6 and Sp8 transcription factors does not prevent the initiation of AER morphology confirming the independence between AER function and morphology. This is in high contrast to *βcatenin* loss-of-function mutants in the limb ectoderm that completely lack any evidence of a morphological AER or ectoderm thickening [14], [15]. This difference may reflect the requirement of βcatenin for a proper AER morphology as has already been suggested [15], [30], [49] and corroborates that the Wnt3/βcatenin-Sp6/Sp8-Fgf8 regulatory loop is not a simple lineal one. Tp63, a crucial factor for AER morphology and *Fgf8* maintenance of expression [61], [62], and a well-established target of Wnt/βcatenin in the ectoderm [73] may be at the root of this difference. Characterization of separate Sp6/Sp8 and Tp63 mediated pathways may help to uncouple βcatenin's multiple roles in AER formation and function.

βcatenin is also necessary for the expression of other AER markers (i.e. *Bmp4*, *Msx2* [14], [15]) in addition to *Fgf8*. *Sp6* and *Sp8* are necessary for the expression of *Fgf8*, but *Bmp* ligands and *Msx2* are normally activated in the total absence of *Sp6/Sp8*. Collectively, these data demonstrate that *Sp6* and *Sp8* mediate only part of the βcatenin functions in the limb ectoderm, principally the induction of *Fgf8*.

Recently, it has been shown that a conserved Wnt-Sp8-Fgf8 genetic cassette is also used to regulate the outgrowth of other body appendages such as the genital tubercle [30]. This work identified Sp8 as partially mediating the regulation of *Fgf8* by the canonical Wnt/βcatenin pathway, a function that we demonstrate here that is fully accomplished by Sp6 and Sp8 together. Their result showing the failure of forced expression of *Sp8* in the AER (*R26Sp8;Msx2*) to rescue the phenotype of *βcatenin* loss-of-function in the limb ectoderm is very likely due to *Sp8* not reaching, in these experiments, the minimum level of expression required for *Fgf8* induction.

### Role of *Sp6* and *Sp8* in dorsal-ventral patterning

During normal development the AER forms at the DV boundary of the limb bud reflecting a tight link between AER formation and DV patterning. Based on the analysis of the *limbless*, *En1* mutants and on misexpression experiments in chick, it was hypothesized that the expression of *En1* in the ventral ectoderm might function to establish a DV interface as a prerequisite for AER induction [74], [75]. However, there are several examples of normal AERs forming in the absence of a DV boundary, such as *eudiplopodia*, the double *Wnt7a;En1* mutant and experiments in chick creating bidorsal limbs [19]–[21], [76].

Here we report that DV patterning is also disrupted when the *Sp6/Sp8* gene dose is perturbed. In the amelic phenotypes, even if the limb does not form, the molecular analysis of the emerging limb buds indicates that they are bi-dorsal as *Wnt7a* expression is extended along most of the limb ectoderm while *En1* is not detected. Interestingly, the failure to activate *En1* occurs despite normal expression of Bmp ligands in the limb ectoderm and mesoderm. In the SHFM phenotypes the digital tips display conical nails. In these limb buds the AER is irregularly induced and where maintained it remains flat, broad and immature. This correlates with an extension of *Wnt7a* expression into the ventral ectoderm and a proximally restricted expression of *En1* [20], [21], [23], [77]. *Lmx1b* expands into the ventral mesoderm distally explaining the bi-dorsal phenotypic traits in the digits of *Sp6^−/−^;Sp8^+/^* ^−^ mutants, while DV patterning is largely preserved at more proximal levels.

We found that in the absence of a sufficient amount of Sp6/Sp8 gene products Bmp signaling is not sufficient to induce *En1*. Sp family members are known to bind and interact with other transcription factors, including Smads. Thus, we hypothesized that Sp6/Sp8 transcription factors interact/cooperate with Smad proteins downstream of Bmp signaling to mediate *En1* activation [78], [79] (Figure 10). This interaction could occur at the protein level or by summative or synergistic effects on the *En1* promoter. Interestingly, the putative *En1* promoter exhibits 25 potential Sp binding sites and 12 Smad binding sites that are conserved between human and mouse. Further investigation will be required to clarify this relationship (Figure S6).

The *Sp6;Sp8* double mutant limbs are reminiscent of those of the *limbless* mutation in chicken. *Limbless* is a simple Mendelian autosomal recessive mutation characterized by tetra-amelia in the homozygous condition [80]. The mutation causes defects in no other organs, although it is effectively lethal because the chicks are unable to hatch without legs [47], [48]. Limb development initiates in *limbless* embryos and the early limb buds are morphologically indistinguishable from normal embryos until stage 19. However, the early limb buds are bidorsal and don't form an apical ridge [74], [75], [81]. The limb bud mesoderm undergoes cell death beginning in the mid-distal mesoderm at stage 19–20 so that by stage 24, no signs of limb buds remain [47], [75]. The similarities between our mutant and the *limbless* mutation may indicate a common target gene. After chromosomal mapping of the *limbless* mutation, Robb and coworkers [82] suggested *Sp8* as a priority candidate. This is reinforced by the fact that *Sp6* seems to be absent in chickens (Figure S3). However, further studies to validate this suspicion have not been done. Interestingly, *limbless* does not display the neural phenotype characteristic of *Sp8* mutants, i.e., except for the limb phenotype the embryo is normal. This could be explained by a defect in a limb specific *Sp8* regulatory element in *limbless*. However, the lack of any AER morphology in *limbless*, in contrast to the double *Sp6;Sp8* mutants, decreases the likelihood that *Sp8* is the gene targeted.

### *Sp6* and *Sp8* and split hand/foot malformation

In humans, the SHFM is a genetically heterogeneous congenital malformation characterized by a deficit in the formation of the central elements of the hands and feet that results in a central cleft associated with fusion and malformations of the remaining digits. The phenotype is highly variable, even between the limbs of a single affected individual, and ranges from a mild central syndactyly to severe loss of elements with oligodactyly and sometimes even affecting the zeugopod. It is currently accepted that this phenotype is the result of a premature regression of the central part of the AER [52], [53], [59]. Remarkably, the limb phenotype of the embryos that develop with a single copy of *Sp8* reproduces the human SHFM condition. The molecular analysis of these mutant limb buds indicates that the product obtained from one allele of *Sp8*, in the absence of *Sp6*, barely reaches the threshold required for *Fgf8* induction. This is based on the low levels of *Fgf8* transcription achieved and also on the irregular expression domain that likely results from a cell autonomous effect of the mutation. Due to normal biologic variation, the level of Sp8 attained may reach the threshold required for *Fgf8* induction in some cells, but not in others. Interestingly, at later stages *Fgf8* expression is not maintained in central regions suggesting that this later deficit in *Fgf8* expression is the cause of the SHFM phenotype in *Sp6;Sp8* compound mutants. Since the irregular early activation of *Fgf8* has not been observed in other models of SHFM, its possible contribution to the phenotype remains to be investigated [52], [53], [59].

Removal of all known AER-related Bmp ligands (*Bmp2*, *Bmp4* and *Bmp7*) from the AER using *Msx2Cre* also results in SHFM [71]. However, in *Sp6^−/−^;Sp8^+/−^* mutants, *Bmp4* is still expressed in the remaining AER suggesting that this SHFM phenotype is not caused by the loss of Bmp expression in the AER. In fact, since Bmp signalling is required for the induction of *Fgf8*, the SHFM phenotype following AER-related Bmp removal can also be explained by an irregular induction of *Fgf8*.

Of great interest is the recent genetic analysis of *Fgf8* regulation that has identified nearly 50 *Fgf8*-regulatory modules in a 220 Kb region centromeric to the gene [83]. All the AER-specific enhancers, many of them embedded in the FBXW4 gene, drive expression all along the AP extension of the AER. Interestingly, SHFM type III [84], [85] is caused by duplications of this genomic region that disrupt the normal architecture of the multiple enhancers likely affecting *Fgf8* expression [83]. Therefore, SHFM type III is likely the result of *Fgf8* misregulation [83].

As previously mentioned, despite the identification of 6 loci involved in SHFM, only *TP63* (SHFM type IV) and *DLX5* and *DLX6* (SHFM type I) have been unequivocally associated with this malformation [34]. Mutations in *WNT10B* (SHFM type VI) were also identified to be causative for SHFM, although there is some doubt on whether these mutations are sufficient for the phenotype [86]–[88]. Since similar phenotypes are frequently caused by disruption of different components of a regulatory network, we have considered the possibility that *Sp6* and *Sp8* genes might be part of the Tp63 network. Indeed, the phenotypes of our mutants are identical, including the DV component, to those recently reported in a new identified human mutation in *DLX5* [57]. However, the fact that *Tp63*, *Dlx5* and *Dlx6* have essentially normal expression patterns in the early *Sp6/Sp8* mutant limb bud indicates that, if Sp6/Sp8 transcription factors act within the Tp63 network, they function downstream of Tp63 and Dlx factors. Tp63 is necessary for the formation and maintenance of a normal epidermal layer [61], [62]. In mouse, removal of *Tp63* results in several abnormalities including limb truncations that are most similar to the *Sp8*-null phenotype [24], [25], [61], [62] suggesting that Tp63 may preferentially control *Sp8*, but not *Sp6* in mice. In any case, the relationship between the Tp63-Dlx and the Sp-Fgf8 regulatory modules, both downstream of Wnt/βcatenin, add an extra level of complexity to limb development that requires further investigation.

### Conclusions

This study provides compelling evidence for the absolute requirement of *Sp6* and *Sp8* for limb development as in their complete absence, or substantial reduction, no limbs form. By using a variety of loss-of-function alleles to remove the activity of *Sp6* and *Sp8* genes, we reveal that these two factors work together and in a dose-dependent manner as necessary mediators for AER development and DV patterning.

Our study supports a model in which these two factors work together downstream of Wnt/βcatenin signaling in the induction of *Fgf8* and also downstream of Bmp signaling in the induction of *En1* establishing a link between proximal-distal and dorsal-ventral patterning.

## Materials and Methods

### Ethics statement and mouse strains

All animal procedures were conducted accordingly to the EU regulations and 3R principles and reviewed and approved by the Bioethics Committee of the University of Cantabria. Mutant mouse lines were described previously: *Sp6* null allele [28]; *Sp8* null allele [24]; *Sp8* floxed allele [36]; *AP2αCre* [39] and *Msx2Cre* lines [37]; R26R [42]. Mice and embryos were genotyped by PCR, using genomic DNA extracted from tail biopsies and yolk sacs, respectively.

### Skeletal preparation

After removing skin and viscera, mouse embryos were fixed in 95% ethanol. Alizarin Red and Alcian blue skeletal staining was performed according to standard protocols, cleared by KOH treatment and stored in glycerol.

### In situ hybridization

In situ hybridization (ISH) was performed in whole-mount and in sections following standard procedures using the previously described *Bmp4* [64], *Dlx5* and *Dlx6* [53], *En1* [23] *Fgf8* [43], *Fgf10* [89], *Lmx1b* [90], *Msx2* [91], *Tp63* [62], *Sp6* [28] and *Wnt7a* [92] antisense riboprobes.

### RNA quantification by real-time PCR

Embryonic fore and hind- limb buds were dissected in cold RNAse-free PBS from E10.5 wild type embryos. Total RNA was isolated separately from 3 pools of 8 forelimbs or 8 hindlimbs each. cDNA synthesis was done using standard conditions.

Real-time RT-PCR was carried out on an Mx3005P cycler, using the SYBRGreen PCR Master Mix (Invitrogen) and the data were analyzed using the MxPro software (Stratagene). Results were tested statistically performing ANOVA and Student-T test, being statistically significant when p<0.05.

Expression of *Sp6* and *Sp8* was normalized to that of housekeeping gene 18sRNA. The primers used (5′ to 3′ orientation) were: Sp6-F: tgctaaccgctgtctgtgg; Sp6-R:ctggtatgtctggagaggttgc; Sp8-F: ttatctccaaggtgcacacg; Sp8-R:gcttgaaccaggactcatacg; 18sRNA-R: ttggcaatgtttcgctc;18sRNA-F: cgccgctagaggtgaaattt.

### Cell death assay

Detection of cell death was performed in sections of paraffin embedded tissue using terminal deoxynucleotidyl transferase mediated dUTP nick-end labelling (TUNEL) with the Apoptag Fluorescein Direct In Situ Apoptosis Detection Kit (Intergen) following the manufacturer's instructions.

### β-gal reporter analysis

For detection of β-galactosidase activity, *R26R;Ap2αCre* double transgenic embryos were fixed for 30 min, rinsed in PBS and incubated in the presence of X-gal as described [93].

### Immunohistochemistry

Immunohistochemistry was performed in paraffin sections using the anti E-cadherin (Byoscience, # 610182), anti Laminin (Abcam, # ab11575), anti Tp63 (Abcam, # Ab53039) and anti Connexin43 (Abcam, # ab11370) primary antibodies. Antigen retrieval was performed by incubation with proteinase K (10 µg/ml) for E-cadherin and laminin or with citrate buffer in pressure cooker for Tp63 and Connexin43. Alexa®488 and TexasRED fluorescently tagged secondary antibodies were used. Vecthasield containing DAPI for nuclear counter staining was used as mounting medium. Confocal images were acquired in a SP-5 laser-scan confocal microscope (Leica Microsystems).

### *In silico* analysis

Conservation of *En1*, *Sp6* and *Sp8* loci between mouse, human, opossum, chicken and zebrafish was determined using pairwise alignment software (mVista browser, http://genome.lbl.gov/vista/). Conserved noncoding regions were further analyzed for potential transcription factor binding sites using AliBaba 2.1 (http://www.generegulation.com/pub/programs/alibaba2/index.html) and Sequencher 4.8 (Gene Codes Inc.) informatic software.

## Supporting Information

Figure S1 Expression of *Sp6* in the limb ectoderm of *Sp8* mutants. Whole mount in situ hybridization for *Sp6* in limb buds of *Sp8* mutant and control littermates. Stage and genotypes as indicated.
(TIF)

Figure S2 Pelvic girdle morphology in *Sp6;Sp8* mutants. Caudal body skeletal preparations of newborns. Genotypes indicated on the left. In the complete absence of *Sp6* and *Sp8*, the pelvis is reduced to a small rudimentary cartilage element. One single functional allele of *Sp6* (*Sp6+/−;Sp8−/−*) leads to the formation of a misshaped ileum and ischium. A schematic drawing showing the three hip bones in different colors (pubis: yellow; ischium: orange and ileum: brown) accompanies each figure.
(TIF)

Figure S3 Analysis 5′ upstream of *Sp6* and *Sp8* (putative promoter regions). Multiple pairwise alignments of the *Sp6* (A) and *Sp8* (B) loci comparing human and the species indicated. Light blue corresponds to the untranslated regions of the gene, dark blue to the coding sequence and pink to noncoding regions with at least 70% conservation. Note that only a portion of the chicken *Sp6* coding sequence is present in Genebank. Conserved regions within the first intron and the region 5′ to the transcription start site containing binding sites are enclosed in red boxes (numbered 1–5 or 1–3, respectively). These conserved regions are illustrated (5′→3′) below the mVista analysis as lines (the actual size is noted above each illustration) and depict the relative positions of potential transcription factor binding sites (see legend within the figure). The motifs used to identify potential binding sites are shown in the boxed insert [94].
(TIF)

Figure S4 Cre reporter activity under the Ap2α locus in the pre-limb ectoderm. (A) Lateral and (B) dorsal views of E8.5 embryo showing ROSA26 reporter activity. (C) transversal section of the same embryo at the level indicated in B. ROSA26 activity was detected in the entire ectoderm at E8.5 (A,B), including the pre-limb ectoderm (black arrowhead in C) and also in the dorsal neural tube.
(TIF)

Figure S5 *AP2αCre* removal of *Sp8* on an *Sp6* deficient background. The external aspect (A, D, G) and skeletal preparations of the forelimb (B, E, H) and hindlimb (C, F, I) of newborns are shown for each genotype (genotypes indicated at the top). Note that the phenotypes are similar to those of the ubiquitous deletions shown in Figure 1. Abbreviations as in Figure 1.
(TIF)

Figure S6 Analysis 5′ upstream of *En1* (putative promoter region). Multiple pairwise alignments of the *En1* locus comparing human and the species indicated. Light blue corresponds to the untranslated regions of the gene, dark blue to the coding sequence and pink to noncoding regions with at least 70% conservation. Conserved regions within the first intron and the region 5′ to the transcription start site containing binding sites are enclosed in red boxes (numbered 1–3). These conserved regions are illustrated (5′→3′) below the mVista analysis as lines (the actual size is noted above each illustration) and depict the relative positions of potential transcription factor binding sites (see legend within the figure). The motifs used to identify potential binding sites are shown in the boxed insert [94].
(TIF)
