# Supplementary figures and images for: Sp6 and Sp8 Transcription Factors Control AER Formation and Dorsal-Ventral Patterning in Limb Development
Source: PLoS Genet. 2014 Aug 28;10(8):e1004468. doi: 10.1371/journal.pgen.1004468 (PMC4148220; doi:10.1371/journal.pgen.1004468)

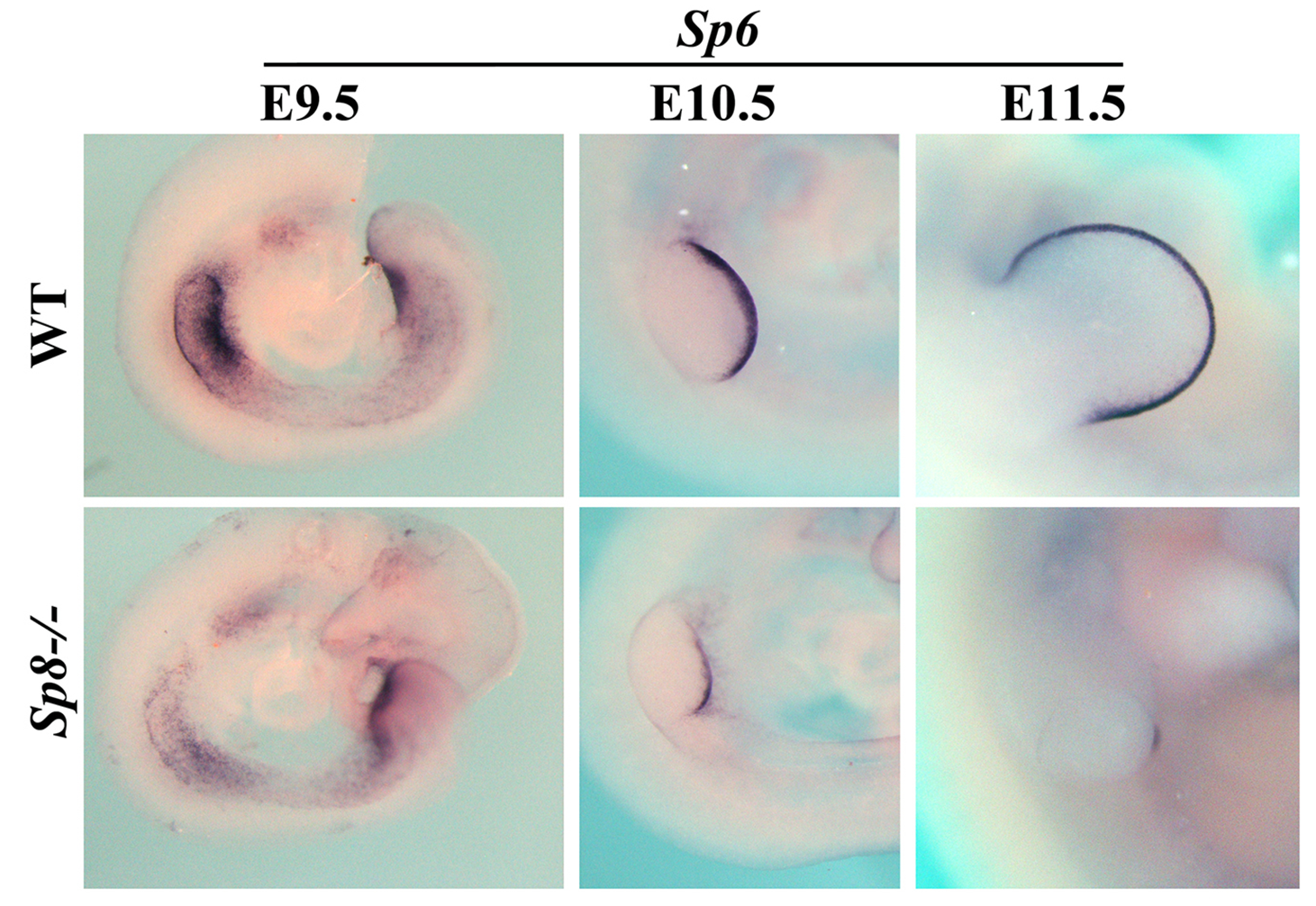

Supplement: Figure S1 — Expression of Sp6 in the limb ectoderm of Sp8 mutants. Whole mount in situ hybridization for Sp6 in limb buds of Sp8 mutant and control littermates. Stage and genotypes as indicated. (TIF) [file pgen.1004468.s001.tif]

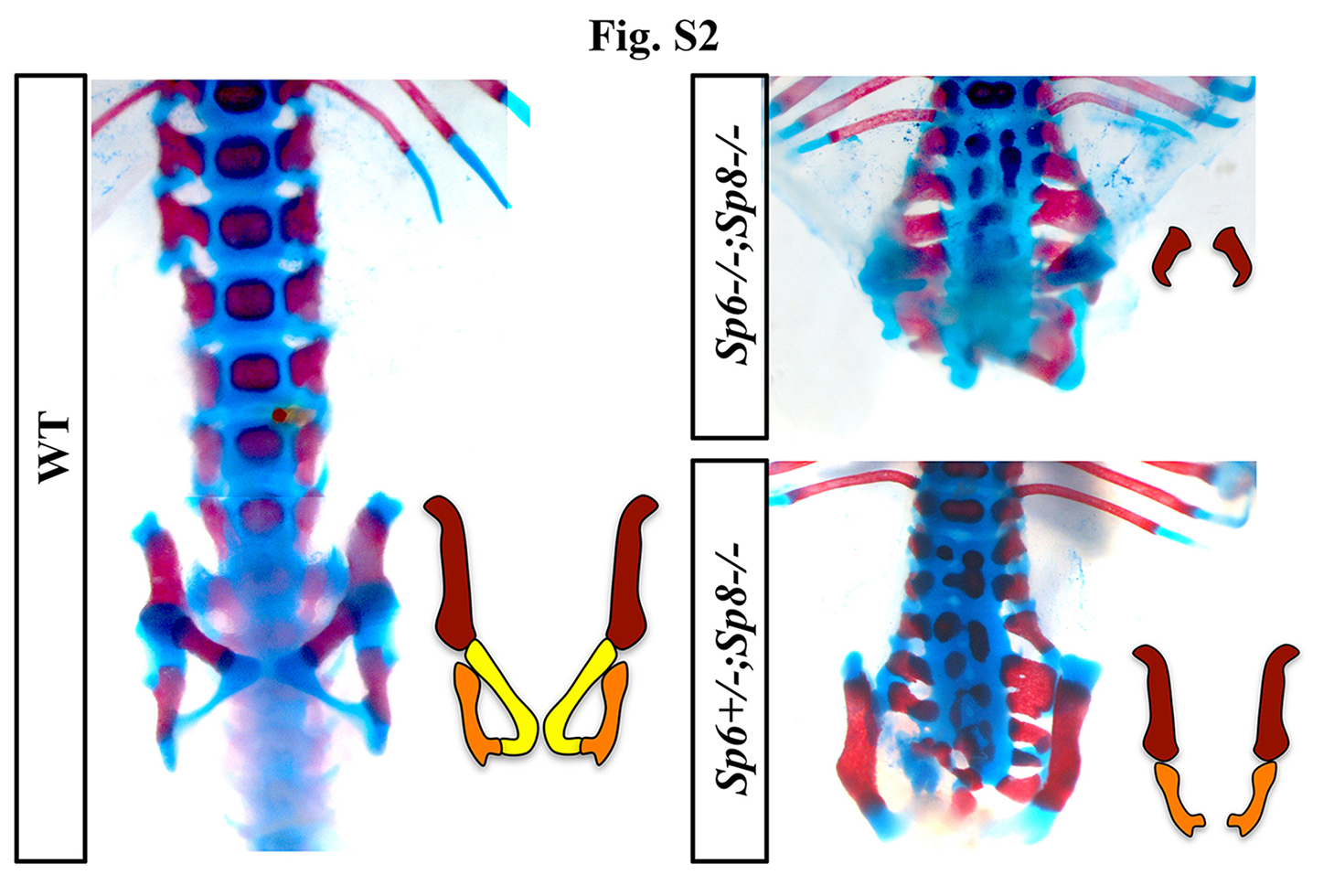

Supplement: Figure S2 — Pelvic girdle morphology in Sp6;Sp8 mutants. Caudal body skeletal preparations of newborns. Genotypes indicated on the left. In the complete absence of Sp6 and Sp8, the pelvis is reduced to a small rudimentary cartilage element. One single functional allele of Sp6 (Sp6+/−;Sp8−/−) leads to the formation of a misshaped ileum and ischium. A schematic drawing showing the three hip bones in different colors (pubis: yellow; ischium: orange and ileum: brown) accompanies each figure. (TIF) [file pgen.1004468.s002.tif]

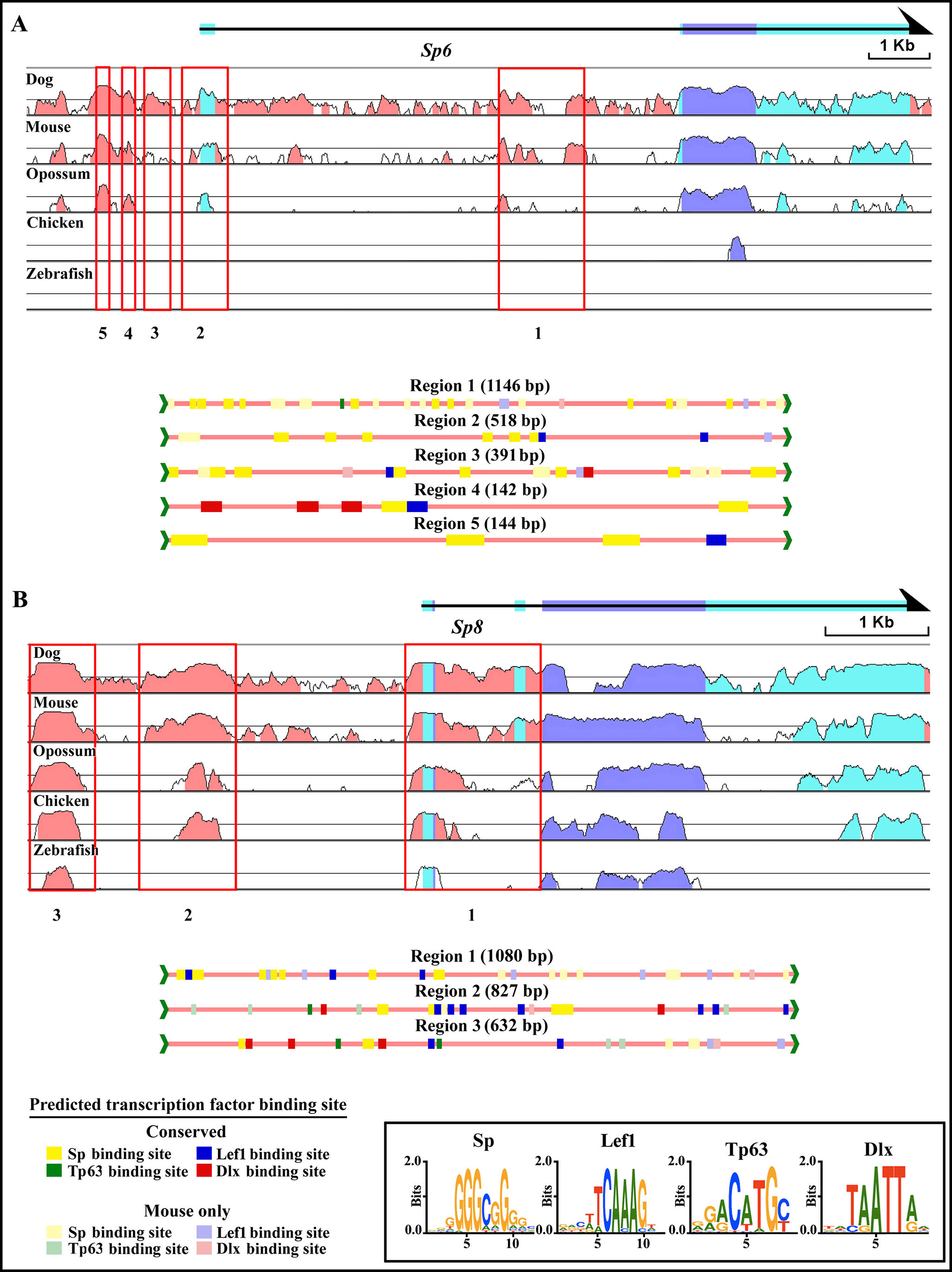

Supplement: Figure S3 — Analysis 5′ upstream of Sp6 and Sp8 (putative promoter regions). Multiple pairwise alignments of the Sp6 (A) and Sp8 (B) loci comparing human and the species indicated. Light blue corresponds to the untranslated regions of the gene, dark blue to the coding sequence and pink to noncoding regions with at least 70% conservation. Note that only a portion of the chicken Sp6 coding sequence is present in Genebank. Conserved regions within the first intron and the region 5′ to the transcription start site containing binding sites are enclosed in red boxes (numbered 1–5 or 1–3, respectively). These conserved regions are illustrated (5′→3′) below the mVista analysis as lines (the actual size is noted above each illustration) and depict the relative positions of potential transcription factor binding sites (see legend within the figure). The motifs used to identify potential binding sites are shown in the boxed insert [94]. (TIF) [file pgen.1004468.s003.tif]

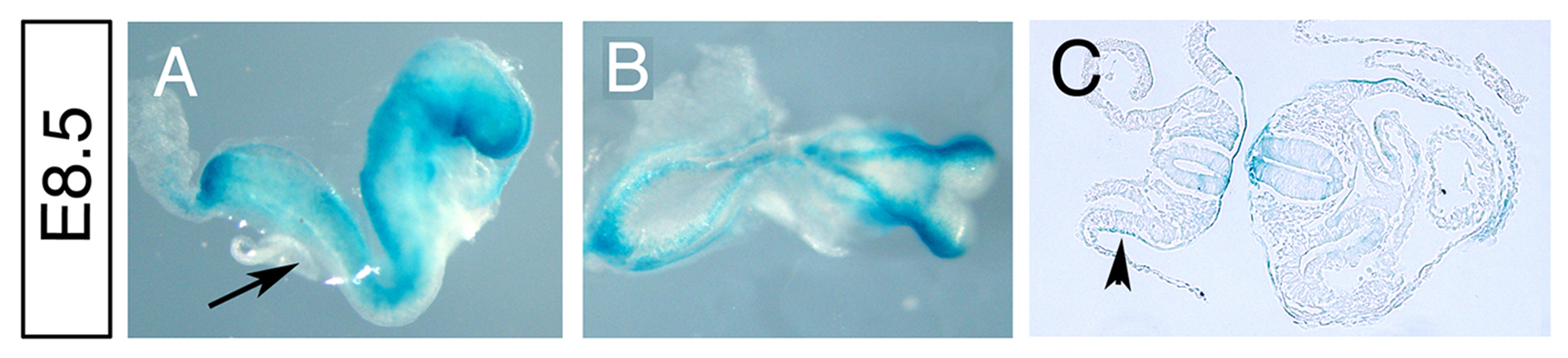

Supplement: Figure S4 — Cre reporter activity under the Ap2α locus in the pre-limb ectoderm. (A) Lateral and (B) dorsal views of E8.5 embryo showing ROSA26 reporter activity. (C) transversal section of the same embryo at the level indicated in B. ROSA26 activity was detected in the entire ectoderm at E8.5 (A,B), including the pre-limb ectoderm (black arrowhead in C) and also in the dorsal neural tube. (TIF) [file pgen.1004468.s004.tif]

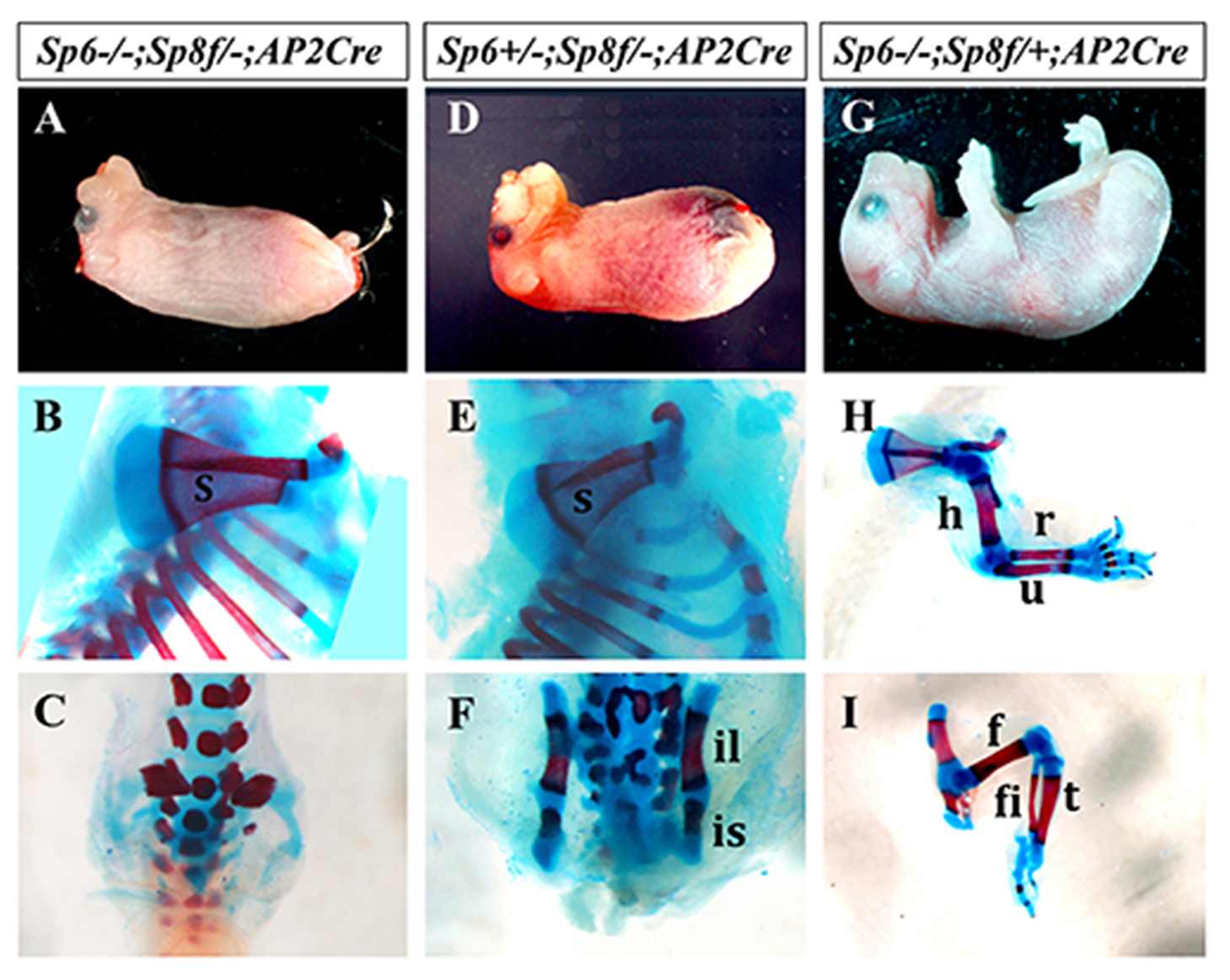

Supplement: Figure S5 — AP2αCre removal of Sp8 on an Sp6 deficient background. The external aspect (A, D, G) and skeletal preparations of the forelimb (B, E, H) and hindlimb (C, F, I) of newborns are shown for each genotype (genotypes indicated at the top). Note that the phenotypes are similar to those of the ubiquitous deletions shown in Figure 1. Abbreviations as in Figure 1. (TIF) [file pgen.1004468.s005.tif]

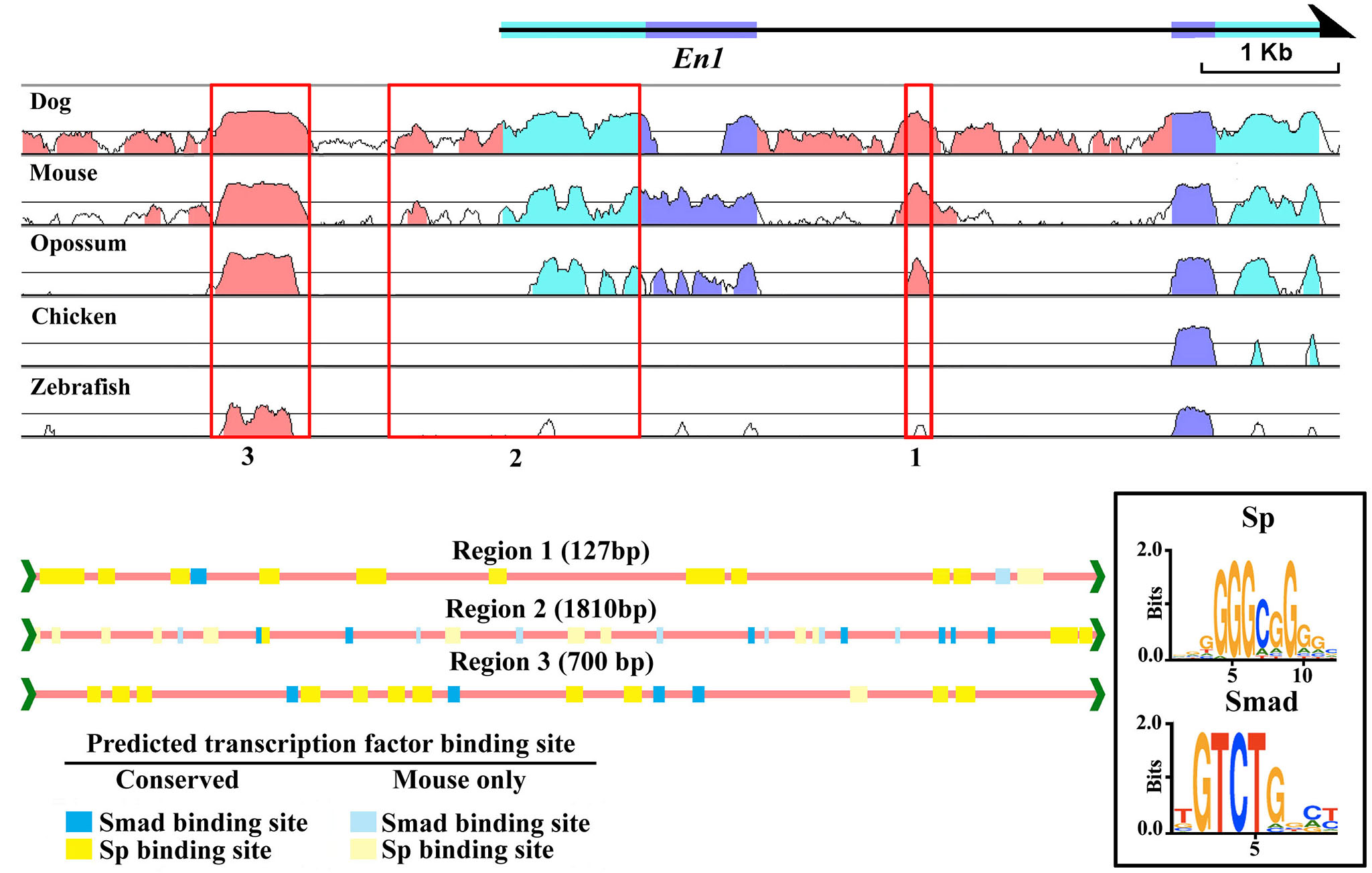

Supplement: Figure S6 — Analysis 5′ upstream of En1 (putative promoter region). Multiple pairwise alignments of the En1 locus comparing human and the species indicated. Light blue corresponds to the untranslated regions of the gene, dark blue to the coding sequence and pink to noncoding regions with at least 70% conservation. Conserved regions within the first intron and the region 5′ to the transcription start site containing binding sites are enclosed in red boxes (numbered 1–3). These conserved regions are illustrated (5′→3′) below the mVista analysis as lines (the actual size is noted above each illustration) and depict the relative positions of potential transcription factor binding sites (see legend within the figure). The motifs used to identify potential binding sites are shown in the boxed insert [94]. (TIF) [file pgen.1004468.s006.tif]
